# The effectiveness and cost-effectiveness of resuscitative endovascular balloon occlusion of the aorta (REBOA) for trauma patients with uncontrolled torso haemorrhage: study protocol for a randomised clinical trial (the UK-REBOA trial)

**DOI:** 10.1186/s13063-022-06346-1

**Published:** 2022-05-12

**Authors:** Jan O. Jansen, Claire Cochran, Dwayne Boyers, Katie Gillies, Robbie Lendrum, Sam Sadek, Fiona Lecky, Graeme MacLennan, Marion K. Campbell, the UK-REBOA Trial grantholders, Jan O. Jansen, Marion K. Campbell, Chris Moran, Karim Brohi, Fiona Lecky, Robbie Lendrum, Graeme MacLennan, Jonathan J. Morrison, Nigel Tai, Tim Harris, John Norrie, Dwayne Boyers, Alan Paterson, Nick Welch

**Affiliations:** 1 Health Services Research Unit, University of Aberdeen, Aberdeen, UK; 2 Department of Surgery, Center for Injury Science, University of Alabama at Birmingham, 1808 7th Ave S, Birmingham, AL 35294 USA; 3 Health Economics Research Unit, University of Aberdeen, Aberdeen, UK; 4 Barts Health NHS Trust, Royal London Hospital, St. Bartholomew’s Hospital, London, UK; 5 Centre for Urgent and Emergency Care Research, Health Services Research Section, School of Health and Related Research, University of Sheffield, Sheffield, UK

**Keywords:** Resuscitative endovascular balloon occlusion of the aorta, REBOA, Trauma, Injury, Resuscitation

## Abstract

**Background:**

Haemorrhage is the most common cause of preventable death after injury. REBOA is a novel technique whereby a percutaneously inserted balloon is deployed in the aorta, providing a relatively quick means of temporarily controlling haemorrhage and augmenting cerebral and coronary perfusion, until definitive control of haemorrhage can be attained. The aim of the UK-REBOA trial is to establish the clinical and cost-effectiveness of a policy of standard major trauma centre treatment plus REBOA, as compared with standard major trauma centre treatment alone, for the management of uncontrolled torso haemorrhage caused by injury.

**Methods:**

Pragmatic, Bayesian, group-sequential, randomised controlled trial, performed in 16 major trauma centres in England. We aim to randomise 120 injured patients with suspected exsanguinating haemorrhage to either standard major trauma centre care plus REBOA or standard major trauma centre care alone. The primary clinical outcome is 90-day mortality. Secondary clinical outcomes include 3-h, 6-h, and 24-h mortality; in-hospital mortality; 6-month mortality; length of stay (in hospital and intensive care unit); 24-h blood product use; need for haemorrhage control procedure (operation or angioembolisation); and time to commencement of haemorrhage control procedure (REBOA, operation, or angioembolisation). The primary economic outcome is lifetime incremental cost per QALY gained, from a health and personal social services perspective.

**Discussion:**

This study, which is the first to randomly allocate patients to treatment with REBOA or standard care, will contribute high-level evidence on the clinical and cost-effectiveness of REBOA in the management of trauma patients with exsanguinating haemorrhage and will provide important data on the feasibility of implementation of REBOA into mainstream clinical practice.

**Trial registration:**

ISRCTN16184981

**Supplementary Information:**

The online version contains supplementary material available at 10.1186/s13063-022-06346-1.

## Administrative information

Note: the numbers in curly brackets in this protocol refer to SPIRIT checklist item numbers. The order of the items has been modified to group similar items (see http://www.equator-network.org/reporting-guidelines/spirit-2013-statement-defining-standard-protocol-items-for-clinical-trials/).

**Table:** 

Title {1}	The effectiveness and cost-effectiveness of Resuscitative Endovascular Balloon Occlusion of the Aorta (REBOA) for trauma patients with uncontrolled torso hemorrhage
Trial registration {2a and 2b}	ISRCTN16184981
Protocol version {3}	Version 6, 25 November 2021
Funding {4}	National Institute for Health Research (NIHR) Health Technology Assessment (HTA) board)
Author details {5a}	Jan O. Jansen, Health Services Research Unit, University of Aberdeen, UK & Center for Injury Science, Department of Surgery, University of Alabama at Birmingham, USA Claire Cochran, Health Services Research Unit, University of Aberdeen, Aberdeen, UK Dwayne Boyers, Health Economics Research Unit, University of Aberdeen, Aberdeen, UK Katie Gillies, Health Services Research Unit, University of Aberdeen, Aberdeen, UK Robbie Lendrum, Barts Health NHS Trust, Royal London Hospital, St. Bartholomew’s Hospital, London, UK Sam Sadek, Barts Health NHS Trust, Royal London Hospital, St. Bartholomew’s Hospital, London, UK Fiona Lecky, Centre for Urgent and Emergency Care Research, Health Services Research Section, School of Health and Related Research, University of Sheffield, Sheffield, UK Graeme MacLennan, Health Services Research Unit, University of Aberdeen, Aberdeen, UK Marion K. Campbell, Health Services Research Unit, University of Aberdeen, Aberdeen, UK And the UK-REBOA Trial grantholders (listed at end of paper)
Name and contact information for the trial sponsor {5b}	This study is co-sponsored by the University of Aberdeen and NHS Grampian.
Role of sponsor {5c}	The sponsor played no part in study design; collection, management, analysis or interpretation of data.

## Introduction

### Background and rationale {6a}

Haemorrhage is the most common cause of preventable death after injury. Trauma disproportionately affects the young, killing those who might otherwise have lived long and productive lives [1]. It is the leading cause of death for children and adults under the age of 46, accounting for nearly half of all deaths in this age group [2]. Taken together, traumatic injuries account for more years of potential life lost before age 75 than any other cause, including cancer or heart disease [1, 3–5].

The natural history of uncontrolled haemorrhage is of falling cardiac output and hypotension and ultimately failure of compensatory mechanisms with consequent cerebral and myocardial hypoperfusion leading to death [6]. Haemorrhage originating from within the torso is particularly challenging, as bleeding generally cannot be controlled without surgery or angio-embolisation [7–9]. In patients in whom haemorrhage is either unrecognised or torrential, exsanguination and death occur prior to definitive haemostasis [4]. However, when haemorrhage is controlled expeditiously, patients often recover [10].

Temporary aortic occlusion can limit haemorrhage and help to maintain perfusion to the heart and brain and is associated with improved survival [11–13]. An adjunctive intervention to temporarily control haemorrhage is thus conceptually attractive and could reduce the number of haemorrhage-related deaths.

**Fig. 1:**
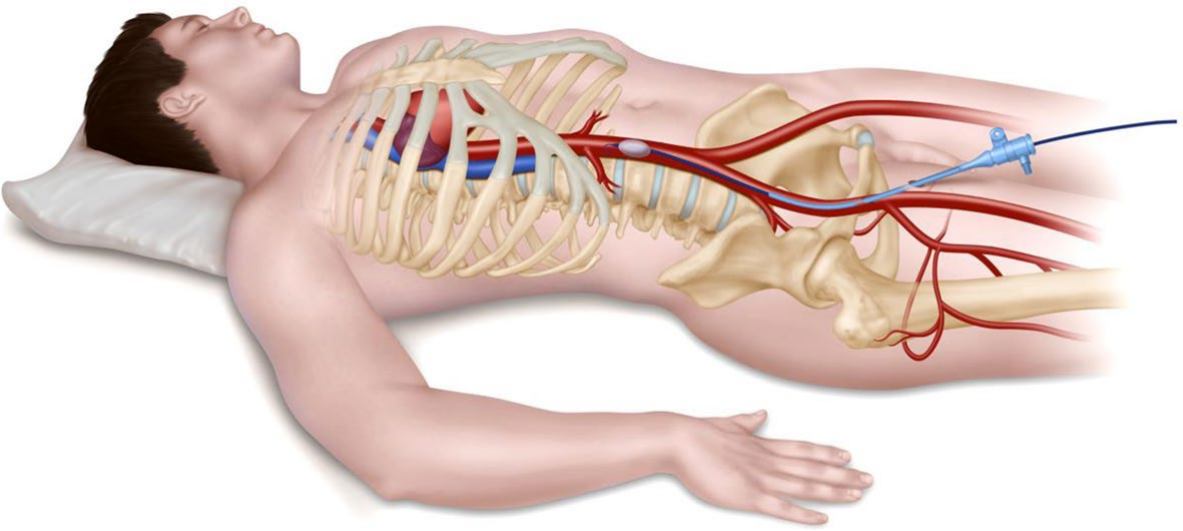
Resuscitative endovascular balloon occlusion of the aorta (REBOA). The device has been inserted into the right common femoral artery and advanced into the abdominal aorta. This placement is used for pelvic haemorrhage. The balloon can also be placed in the thoracic aorta, to control abdominal (with or without pelvic) haemorrhage

REBOA is a novel technique whereby a percutaneously inserted balloon is inflated in the aorta (Fig. 1), providing a relatively quick means of temporarily controlling haemorrhage, by markedly reducing distal blood pressure and therefore blood loss, until definitive control of haemorrhage can be obtained. REBOA increases cardiac afterload and proximal aortic pressure and thus improves perfusion of the heart and brain, and large animal models of uncontrolled haemorrhage have shown REBOA to be highly effective [14–17]. However, REBOA is not without potential risks. Failure to insert the device could waste valuable time that would be better spent taking the patient directly to an operating theatre, to obtain surgical control of bleeding. Insertions may also be associated with major damage to blood vessels. Even if the balloon is successfully deployed, the (intentional) severe reduction in distal blood pressure, unless very short, can result in impaired tissue perfusion, ischaemic damage, or thromboses, which may be irreversible.

The current evidence for REBOA in injured humans is limited and conflicting. There are a number of case series [18–20]; cohort studies (retrospective and prospective) [21–24], with divergent results; and several scoping reviews, systematic reviews, and meta-analyses [25–29]. There are also military clinical practice guidelines [30] and a position statement from the American College of Emergency Physicians and the American College of Surgeons [31]. However, there are no randomised clinical trials.

### Objective {7}

The objective of the UK-REBOA trial is to establish the clinical and cost-effectiveness of REBOA in addition to standard care, as compared with standard care alone, for the management of uncontrolled torso haemorrhage, in specialist major trauma centres.

### Trial design {8}

**Fig. 2:**
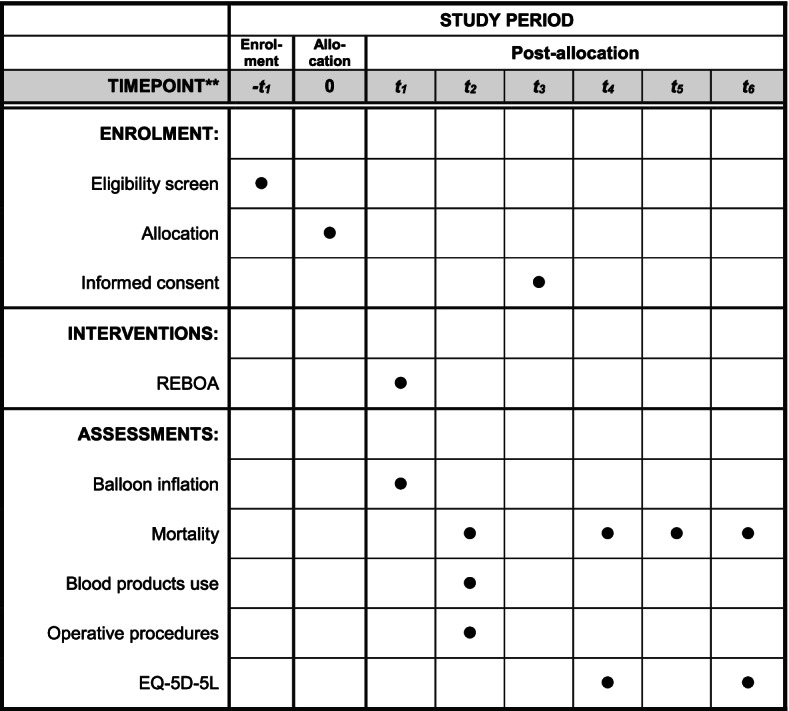
Participant timeline showing schedule of enrolment, interventions, and assessments. t_1_, immediately following allocation (typically minutes); t_2_, within 24 h of allocation; t_3_, on discharge from critical care; t_4_, on discharge from acute care hospital; t_5_, at 90 days post-injury; t_6_, 6 months post-injury

**Fig. 3:**
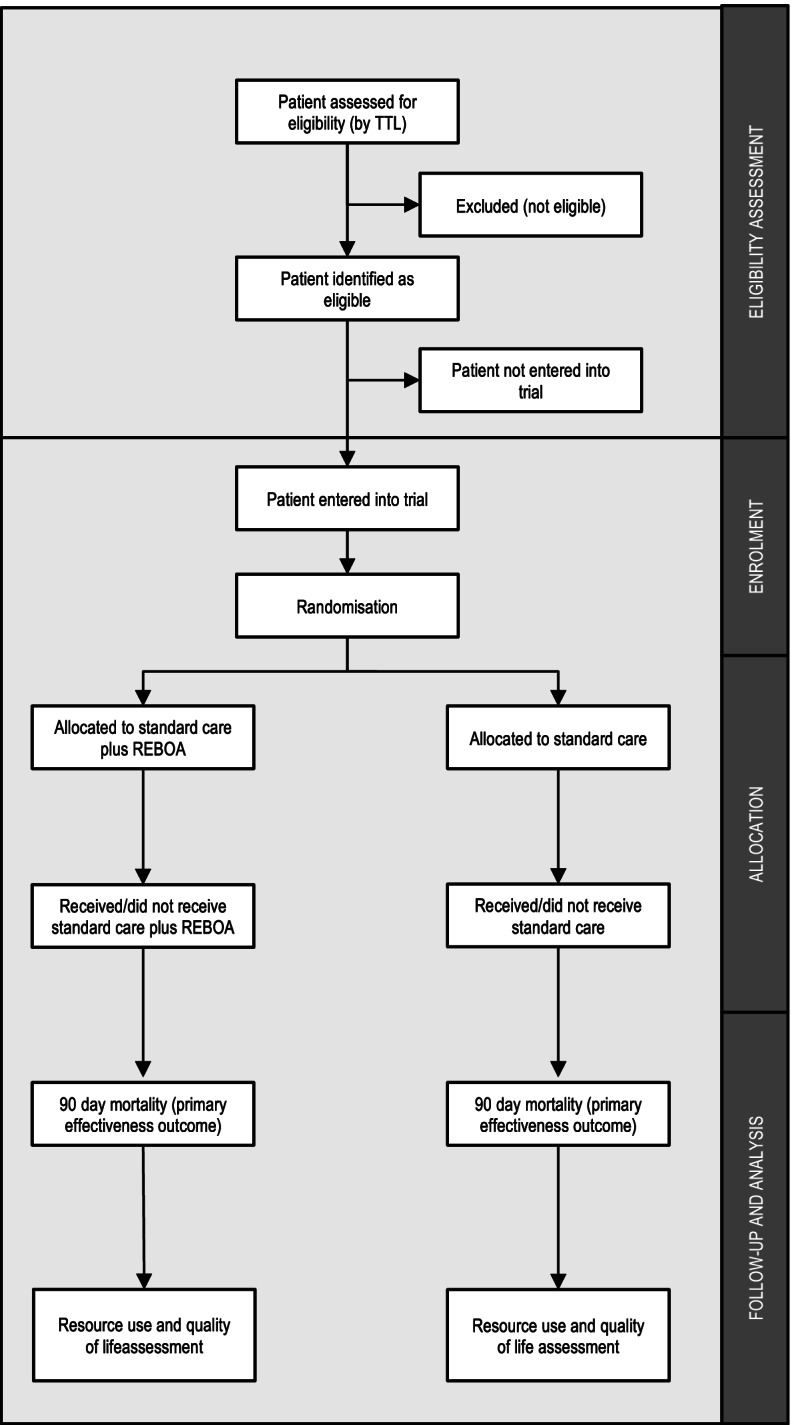
CONSORT diagram

This is a multi-centre, Bayesian, group-sequential, parallel-group randomised controlled trial. This protocol is written in accordance with the “Standard Protocol Items: Recommendations for Interventional Trials (SPIRIT) checklist” (and item numbers are included in curly brackets). The schedule of enrolment, interventions, and assessments for the duration of the study is tabulated in Fig. 2. Patients enrolled in the study are randomised into two arms (Fig. 3).

The trial also includes an embedded mixed-methods process evaluation, using a behavioural science approach to identify challenges to the inclusion of patients and the delivery of the intervention. This process evaluation includes theory informed interviews with staff involved in the trial (randomisation, intervention delivery, etc.) at a range of sites, documentary review of training materials, and observations of training events. The results of this evaluation are to be fed back to sites and included in ongoing local and national training events in real time, in order to support and improve enrollment. This process evaluation is to be reported separately.

## Methods: participants, interventions, and outcomes

### Study setting {9}

**Fig. 4:**
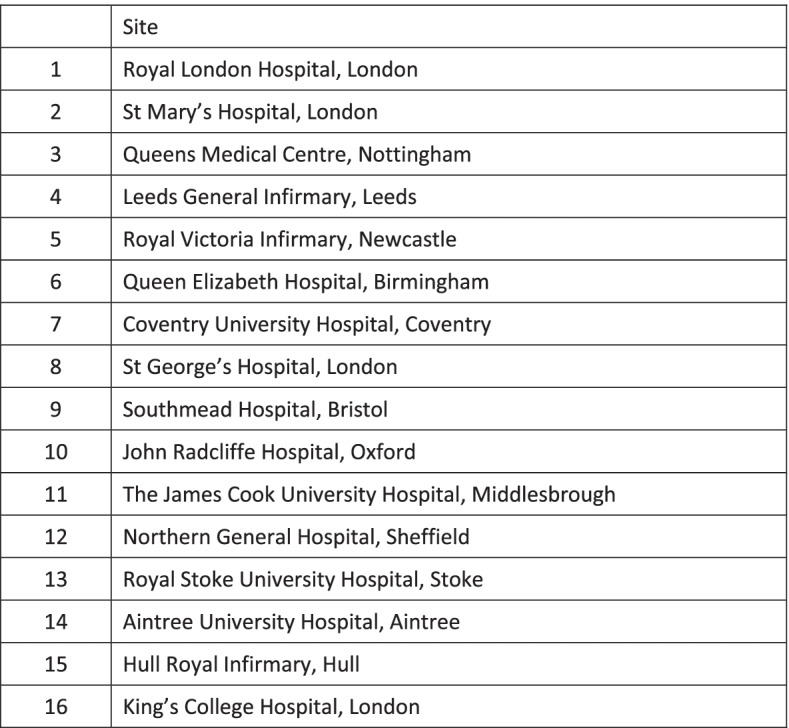
Participating major trauma centres

The trial is being conducted in 16 major trauma centres in England, listed in Fig. 4. Major trauma centres are specialist, tertiary centres designated to provide definitive care for seriously injured patients. Screening, recruitment, and baseline data collection therefore take place in emergency departments. Patients are followed up to discharge from acute care, and virtually (by data linkage) after discharge.

### Eligibility criteria {10}

The trial seeks to enrol trauma patients at imminent risk of dying, from acute blood loss due to torso or junctional haemorrhage, in the emergency department. Patients are eligible if they meet the following criteria: aged, or believed to be aged, 16 years or older or with confirmed or suspected life-threatening torso haemorrhage, which is thought to be amenable to adjunctive treatment with REBOA. These criteria were chosen because they reflect the global assessment which clinicians intuitively perform when evaluating severely injured patients, and the pressured clinical setting in which this research has to be conducted. Women known or thought to be pregnant at presentation and patients with injuries that are deemed clinically unsurvivable will be excluded.

### Who will take informed consent? {26a}

Patients who are eligible for inclusion in the trial are incapacitated and unable to give consent at the time of eligibility assessment and randomisation. Similarly, there is not sufficient time to consult a surrogate decision-maker, or even an independent medical practitioner, for advice about including the patient. Enrolment therefore takes place without consent. There is legal provision and precedent [32] for conducting research in these circumstances, in England, in the form of the Mental Capacity Act (2005) (for non-CTIMP trials). Consent is subsequently sought by a member of the treating clinical team after the patient has been enrolled, and treatment has been completed, but at this stage will relate to follow-up and future contacts, electronic tracing, and data linkage, rather than the intervention itself. Ethics approval for the trial was obtained from the Greater Manchester Research Ethics Committee.

### Additional consent provisions for collection and use of participant data and biological specimens {26b}

The trial does not involve the collection of biological specimens.

## Interventions

### Explanation for choice of comparators {6a}

As outlined above, temporary aortic occlusion can limit haemorrhage, can help to maintain perfusion to the heart and brain, and is associated with improved survival. REBOA potentially provides a relatively quick means of achieving this temporary control. Thus, the UK-REBOA trial compares the addition of REBOA to standard care provided when an exsanguinating trauma patient arrives at a major trauma centre.

### Intervention description {11a}

The intervention is the technique of endovascular aortic occlusion, for the purpose of resuscitation, as part of an overall treatment strategy. The addition of REBOA to current treatment is intended to provide earlier, temporary haemorrhage control, to facilitate transfer to an operating room or interventional radiology suite, for definitive haemostasis. The trial seeks to evaluate the technique of REBOA rather than a specific brand of device and therefore permits the use of any licensed occlusion balloon, and does not prescribe or mandate a particular product.

Patients allocated to the control group receive “standard care”, as expected in a specialist major trauma centre. Such treatment typically includes intubation, blood transfusion, and early operative or endovascular haemorrhage control. Treatment can also include open aortic occlusion, of the thoracic or abdominal aorta.

### Criteria for discontinuing or modifying allocated interventions {11b}

In patients who had been randomised to the REBOA arm of the trial, clinicians are at liberty to not insert the balloon occlusion device if the patient’s hemodynamic status improves (either spontaneously, or as a result of ongoing blood transfusions), and they are deemed to no longer have life-threatening torso haemorrhage, amenable to adjunctive treatment with REBOA; deteriorated (to the point of imminent death); or there was technical difficulty in obtaining arterial access, and it was felt that operative control of haemorrhage could be obtained more quickly. Patients are also free to withdraw from the study.

### Strategies to improve adherence to interventions {11c}

There are no strategies to improve adherence, if randomisation is not followed.

### Relevant concomitant care permitted or prohibited during the trial {11d}

Use of REBOA does not require alteration to usual care pathways (including use of any medication) and these will continue for both trial arms. All relevant medical care is permitted.

### Provisions for post-trial care {30}

There is no provision for post-trial care beyond the standard major trauma centre care provided to severely injured patients. Such care may involve multiple specialties, depending on the nature of patients’ injuries.

### Outcomes {12}

#### Primary clinical outcome measure

The primary clinical outcome is 90-day mortality (defined as death within 90 days of injury, before or after discharge from hospital). This outcome is intended to capture any late harmful effects of REBOA.

#### Primary economic outcome measure

The primary economic outcome is lifetime incremental cost per QALY gained, modelled over a lifetime horizon, from a health and personal social services perspective.

#### Secondary clinical outcome measures

Secondary clinical outcomes include 3-h, 6-h, and 24-h mortality; in-hospital mortality; 6-month mortality; length of stay (in hospital and intensive care unit); 24-h blood product use; need for haemorrhage control procedure (operation or angioembolisation); and time to commencement of haemorrhage control procedure (REBOA, operation, or angioembolisation).

#### Secondary economic outcome measures

Secondary economic outcomes include 6-month costs from an NHS and personal social services perspective, quality of life (measured using EQ-5D-5L) at 6 months, and incremental cost per QALY gained at 6 months.

These outcomes were chosen prior to the publication of core outcomes for patients undergoing REBOA [33], and prior to the publication of recommendations regarding the choice of outcomes for haemorrhage control trials [34].

### Participant timeline {13}

The participant timeline is shown in Fig. 2.

### Sample size {14}

We designed the trial around the available number of patients, rather than calculate a minimum sample size required, based on a retrospective study of national Trauma Audit and Research Network (TARN) data [35]. We estimated that 10 high-volume MTCs would admit approximately 80 patients who might benefit from REBOA, per year, approximately half of whom would be enrolled into the trial, and further estimated that we would be able to enrol 120 patients over a period of 3 years, with a staggered start. Actual enrollment rates in early sites were lower than our original estimates, and we therefore added a further six major trauma centres.

### Recruitment {15}

Patients are recruited based on the assessment of the trauma team leader (typically a consultant) in the emergency department. Consent for continuing participation (i.e. data collection) is sought by a member of the UK-REBOA team taking care of the patient at the hospital site once patients are no longer in a critical condition. This is defined as being cared for in a ward area (rather than an intensive care unit or high dependency unit).

## Assignment of interventions: allocation

### Sequence generation {16a}

Patients are enrolled by the doctor in charge of the patient’s care, using a dedicated and secure website, accessible from handheld devices such as smartphones, tablets (one of which is provided to each centre), or a desktop computer in the resuscitation area. This mechanism takes cognisance of the extreme acuity with which eligible patients will present and minimises distraction of the medical team. The website links directly to the randomisation system in the Centre for Healthcare Randomised Trials (CHaRT) at the University of Aberdeen. All trauma team leaders and research staff are issued usernames and passwords for the randomisation website. The website is designed to require minimal data entry, so as not to distract clinicians from delivering life-saving care. Patients are randomised into one of the two intervention arms, in a 1:1 ratio, in randomly generated blocks of two and four, in order to reduce predictability and selection bias.

### Concealment mechanism {16b}

This is an open-label study. There is no concealment. However, the allocation sequence is concealed from the doctor in charge of the patient’s care until they log into the dedicated website, in the Centre for Healthcare Randomised Trials, using their smart phone. They are then notified which intervention arm the patient was being randomised to.

### Implementation {16c}

The randomisation website returns a patient study ID and the patients’ allocation, which is then implemented by clinical teams. Most of the participating sites had not used REBOA previously. The implementation strategy had four components: (1) initial training, which introduced the technique to sites, and trained most of the staff, including future trainers; (2) development of a local service delivery and training framework, for ongoing skill development and training of new staff; (3) reminder training sessions; and (4) commercial product support. Training included technical aspects (the insertion of the device) as well as clinical decision-making, application of the inclusion criteria, ethical considerations, and post-REBOA management of patients.

## Assignment of interventions: blinding

### Who will be blinded? {17a}

As this is an open-labelled study, patients, investigators, and analysts are not blinded.

### Procedure for unblinding if needed {17b}

This is an open-label trial, and there is no need for an unblinding procedure.

## Data collection and management

### Plans for assessment and collection of outcomes {18a}

The data collection strategy for the UK-REBOA trial is designed to minimise the burden on participants and clinicians, and the avoidance of duplication. The trial draws on routinely collected data, from a number of sources, whenever possible, and can be regarded as a registry-enabled randomised controlled trial, although the case identification is not based on the registry, and the linkage occurs later.

#### Trauma Audit and Research Network (TARN) national trauma registry data

Data on the treatment of trauma patients is routinely collected by the Trauma Audit and Research Network (TARN, https://www.tarn.ac.uk), the national trauma registry for England, to which all major trauma centres are required to submit data. TARN collect demographic, injury, treatment, and outcome data, and—through a third-party provider—patient-reported outcome measures (PROMs), including EQ-5D-5L. Data collected by TARN directly is known to be very complete and of high quality [36].

#### NHS Digital Data

In addition to drawing on TARN data, the trial also links to NHS England’s Hospital Episode Statistics (HES) data to obtain information on hospital resource use and to Office of National Statistics (ONS) data for medium-term (6-month) mortality.

#### Mortality

Survival status and, where applicable, date and time of death are recorded in both the TARN and ONS data. However, in order to minimise delays in reporting, we will also obtain death data directly from sites.

#### Duration of balloon inflation and use of partial occlusion

The duration of balloon inflation is important, as prolonged occlusion of the aorta leads to profound distal ischaemia and (if the balloon is deflated), reperfusion injury, which can be fatal. Balloon inflation and deflation times are often inaccurately recorded in clinical practice, and these datapoints are therefore included on the website used to enrol and randomise patients. Once a patient has been randomised to REBOA, a new screen appears on which the time of balloon inflation and final deflation (if there were multiple attempts) can be recorded. In addition, if partial occlusion is used (to allow some blood flow to the lower part of the body), this can also be recorded.

#### EQ-5D-5L

Following the first linkage run, it became clear that the EQ-5D-5L results collected by the third-party provider contracted by TARN were incomplete. We therefore asked sites to collect EQ-5D-5L data prior to discharge, and subsequently at approximately 6 months by telephone.

### Plans to promote participant retention and complete follow-up {18b}

The trial intervention pertains only to inpatient management. Furthermore, the intervention has been completed by the time patients (or consultees) are approached for consent. Retention and follow-up was therefore not anticipated to be problematic. However, patients were encouraged to engage with investigators regarding the collection of post-discharge EQ-5D-5L data.

### Data management {19}

Data are entered directly into eCRFs, housed in CHaRT, in the Health Services Research Unit, at the University of Aberdeen, with off-site backup. Records will be destroyed after 10 years, upon approval from sponsor and ethics committees.

### Confidentiality {27}

Data collected during the course of the research will be kept strictly confidential and only accessed by members of the trial team (or individuals from the sponsor organisation or centre sites where relevant to the trial). Participants are allocated an individual study number upon randomisation. Participants’ details are stored on a password-protected database and only accessible to the study team. Participant’s data is fully anonymised for analysis and reporting.

### Plans for collection, laboratory evaluation, and storage of biological specimens for genetic or molecular analysis in this trial/future use {33}

See above {26b}. There will be no biological specimens collected.

## Statistical methods

### Statistical methods for primary and secondary outcomes {20a}

#### Primary clinical outcome

The number of eligible patients is small, and we have therefore adopted a Bayesian inferential framework for this trial, which has been described in detail in another publication [37]. The primary endpoint is the log odds ratio of 90-day survival after major trauma centre treatment with REBOA, compared to major trauma centre treatment alone:

$$\log \left(\frac{\frac{p_R}{1-{p}_R}}{\frac{p_S}{1-{p}_S}}\right)=\log \left(\frac{p_R\left(1-{p}_S\right)}{p_S\left(1-{p}_R\right)}\right)=\log \left(\frac{p_R}{1-{p}_R}\right)-\log \left(\frac{p_S}{1-{p}_S}\right)=\delta$$

where *p*_*R*_ and *p*_*S*_ are the proportions of patients surviving, to 90 days, after REBOA and standard care, respectively.

Bayesian designs permit the inclusion of prior information about *δ.* The final analysis of the trial will include a range of prior probability distributions, to contextualise the trial’s findings. This approach has been used in a number of recent studies [38, 39]. We anticipate including at least two priors, as well as a neutral non-informative prior with mean *μ*_0_ = 0 and very large prior variance *σ*_0_^2^ (or, equivalently, very small prior precision *τ*_0_ = 1/*σ*_0_^2^) for the control and intervention survival proportions, and also on the treatment effect. Further assumptions are that the treatment effect is normally distributed (on the log-odds scale) with a known variance. These assumptions will allow us to use a normal likelihood for the data; coupled conjugate normal prior distributions will lead to a normal posterior distribution. All decisions about stopping the trial, or declaring success, will be made based on probabilities derived from the posterior distribution of the treatment effect, and a neutral prior. A detailed statistical analysis plan (SAP) will be developed prior to the final analysis.

#### Primary economic outcome

A “within trial” economic analysis will assess and compare costs and outcomes collected for participants enrolled in the trial up to 6 months post-randomisation. These data will then be used to inform key input parameters in an economic model which will be developed to estimate the long-term cost-effectiveness of standard major trauma centre care with REBOA versus standard major trauma centre care alone. A draft economic model was developed during the feasibility stage based on available literature, to help inform and refine the economic data requirements for the main trial phase. The final model-based analysis will assess the incremental cost per QALY gained with REBOA versus standard major trauma centre care over a lifetime horizon and a value of information (VOI) analysis will be used to guide future research prioritisation.

#### Secondary outcomes

Secondary outcomes will also be analyzed using a Bayesian approach, similar to the primary outcome but using the appropriate model for the outcome distribution.

### Interim analyses {21b}

We will conduct two interim analyses, after 40 and 80 randomised participants, and a final analysis after the expected maximum of 120 randomised participants. The trial will be stopped early if the probability that the 90-day survival odds ratio (OR) falls below 1 (i.e. REBOA is harmful) at the first or second interim analysis is 90% or greater. More formally, our Bayesian futility criterion at each stage is *P* (*δ <* 0 | *y*) ≥ 0*.*9 where *δ* is the log OR and *y* is the observed data. REBOA will be declared “successful” if the probability that the 90-day survival OR exceeds 1 at the final analysis is 95% or greater, so our Bayesian success criterion is defined as *P* (*δ >* 0 | *y*) ≥ 0*.*95. Our calculations are based on an estimated control group (standard major trauma centre treatment alone) 90-day survival rate of 66.5% [35].

### Methods for additional analysis (e.g. subgroup analyses) {20b}

We do not plan to conduct additional or subgroup analyses.

### Methods in analysis to handle protocol non-adherence and any statistical methods to handle missing data {20c}

We do not anticipate missing data for the primary outcome. The primary analysis will be by intention to treat.

### Plans to give access to the full protocol, participant-level data, and statistical code {31c}

Formal requests for access to anonymised study data and/or to the statistical code will be considered on a case-by-case basis by the trial management group.

## Oversight and monitoring

### Composition of the coordinating centre and trial steering committee {5d}

#### Coordinating centre

The study is led by the Centre for Healthcare Randomised Trials in the Health Services Research Unit at the University of Aberdeen. The Project Management Group consists of the two co-Chief Investigators, a Trial Manager, a Senior Trial Manager, and a Data Coordinator.

#### Trial steering committee

The trial is overseen by an independent Trial Steering Committee (TSC), which includes two patient/public representatives. The TSC meets at least annually. The TSC adheres to a charter which is available upon request to the lead author.

### Composition of the data monitoring committee, its role, and reporting structure {21a}

The trial is monitored by an independent data monitoring committee (DMC). The DMC meets at least annually and reports to the trial steering committee. The DMC adheres to a charter which is available upon request to the lead author.

### Adverse event reporting and harms {22}

As this study is recruiting in trauma patients with life-threatening injuries, it is expected that many of the patients will experience events that are the consequence of the patient’s life-threatening injuries, resulting critical illness, and treatment. All adverse events/device effects occurring between randomisation and discharge are recorded in the appropriate case report form and closely monitored by the oversight committees.

#### Expected complications

Death and a number of expected complications (including some which result in life-threatening illness, permanent impairment of structure or function, additional medical or surgical intervention, or prolonged hospital stay) are pre-specified outcomes and will therefore not be reported as serious adverse events (SAEs) or serious device adverse events (SADEs). Only unexpected SAEs/SADEs will be reported to the sponsor.

The following adverse events can be expected to occur as a result of using REBOA. Access-related adverse device effects (ADEs): external haemorrhage at insertion site requiring treatment other than simple pressure, pseudoaneurysm, arteriovenous fistula, dissection of artery, extremity ischaemia, stenosis of artery, distal embolism, air embolism, infection requiring surgical intervention, need for patch angioplasty (surgical repair), need for arterial bypass, and need for amputation. Other adverse device effects (ADEs) are balloon rupture, aortic rupture, and side branch cannulation.

#### Adverse events related to standard treatment

The following adverse events can be expected to occur as a result of standard aortic occlusion, by means of a thoracotomy or laparotomy. Adverse events (AEs) related to external thoracic aortic occlusion are descending thoracic aortic injury, lung injury/bronchopleural fistula, cardiac injury, oesophageal injury, empyema, wound infection requiring surgical intervention, sternal non-union, rib fractures, extremity ischaemia, distal embolism, infection requiring antibiotics only, and infection requiring surgical intervention. Adverse events (AEs) related to external abdominal aortic occlusion are abdominal aortic injury, wound infection requiring surgical intervention, extremity ischaemia, distal embolism, infection requiring antibiotics only, and infection requiring surgical intervention.

#### Adverse events common to both treatments

Adverse events (AEs) related to impaired organ perfusion are acute kidney injury requiring renal replacement therapy, mesenteric ischaemia requiring surgical intervention, paraplegia (permanent), paraplegia (temporary), acute respiratory distress syndrome, stroke (embolic or hypoperfusion-related), and multi-organ failure.

#### Adverse event/device effect reporting

The principal investigator (PI) at each site or their delegated investigator is responsible for recording and reporting of AEs/ADEs observed during the study period. The PI must assess severity, seriousness, causality, and expectedness for any AEs/ADEs in keeping with regulatory requirements. The investigator should attempt, if possible, to establish a diagnosis based on the participant’s signs and symptoms. When a diagnosis for the reported signs or symptoms is known, the investigator should report the diagnosis as the AE/ADE, rather than reporting the individual symptoms.

#### Serious adverse event/device effect reporting

All events meeting the definition of a serious adverse event (SAE) or serious adverse device effect (SADE) will be entered onto the Serious Adverse Event/Serious Adverse Device Event reporting form and submitted to CHaRT within 24 h of the investigator becoming aware of the event. The PI at the site should not wait until all information about the event is available before notifying CHaRT of an SAE/SADE. Information not available at the time of the initial report must be documented on a follow-up SAE/SADE Form. Follow-up information should be sought and submitted as it becomes available. The follow-up information should describe whether the event has resolved or persists, if and how it was treated, and whether the patient continues on the study or has been withdrawn from treatment. Once received, seriousness, causality, and expectedness will be confirmed by the Chief Investigator (or delegated clinical lead). Unanticipated serious adverse device effects (USADE): SAEs that are deemed to be related to the study device or any of the research procedures and are unanticipated will be notified to the sponsor and Research Ethics Committee (REC) within 15 days of CHaRT becoming aware of the event.

#### Grading of severity of adverse events

The PI or designee will assess the severity for each AE using the following standard criteria. (1) Mild: The adverse event/device effect does not interfere with the participant’s daily routine and does not require intervention; it causes slight discomfort. (2) Moderate: The adverse event/device effect interferes with some aspects of the participant’s routine, or requires intervention, but is not damaging to health; it causes moderate discomfort. (3) Severe: The adverse event/device effect results in alteration, discomfort, or disability which is clearly damaging to health. (4) Life threatening: An adverse event/device effect that has life threatening consequences; urgent intervention indicated. (5) Fatal: An adverse event/device effect that results in death.

#### Assessment of seriousness

The PI or designee should make an assessment of seriousness. As stated above, death and a number of expected complications (including some which result in life-threatening illness, permanent impairment of structure or function, additional medical or surgical intervention, or prolonged hospital stay) are pre-specified outcomes and will therefore not be reported as SAEs/SADEs.

#### Assessment of causality

The PI or designee should make an assessment of the causality (i.e. relationship to trial device) for each event. Events which are possibly, probably, or definitely related to the device are reported as related. This will be determined as follows: (1) Definitely: There is clear evidence to suggest a causal relationship, and other possible contributing factors can be ruled out. (2) Probably: There is evidence to suggest a causal relationship, and the influence of other factors is unlikely. (3) Possibly: There is some evidence to suggest a causal relationship (e.g. the event occurred within a reasonable time after using the device). However, the influence of other factors may have contributed to the event (e.g. the patient’s clinical condition, other concomitant events). (4) Unlikely: There is little evidence to suggest there is a causal relationship (e.g. the event did not occur within a reasonable time after administration of the trial medication). There is another reasonable explanation for the event (e.g. the patient’s clinical condition, other concomitant treatments). (5) Not related: There is no evidence of any causal relationship. (6) Not assessable: Unable to assess on information available.

#### Assessment of expectedness

The PI or designee should make an assessment of expectedness for each SAE/SADE regardless of the causal relationship to the trial device.

#### Follow-up procedures

All AEs/ADEs assessed by the PI or designee as possibly, probably, or definitely related to the device and all SAEs/SADEs that occur during this time will be followed until they are resolved or are clearly determined to be due to a patient’s stable or chronic condition or intercurrent illness(es). The CRF should be updated with the date and time of resolution or confirmation that the event is due to the patient’s illness as soon as this information becomes available.

#### Recording and reporting of urgent safety measures

If the PI, designee, or a member of study staff becomes aware of information that necessitates an immediate change in study procedure to protect clinical trial participants from any immediate hazard, they should report the urgent safety measure immediately to CHaRT. CHaRT will report the urgent safety measure immediately to the sponsor and will liaise with the sponsor and site to implement immediate procedures to eliminate any hazard. CHaRT will report immediately by phone to the study REC and will follow this up with an email written notice within 3 days of becoming aware of the urgent safety measure. The email notice will state the reason for the urgent safety measure and the plan for further action. The PI or designee should respond to queries from CHaRT immediately to ensure the adherence to these reporting requirements.

### Frequency and plans for auditing trial conduct {23}

The multidisciplinary project management group meet twice a month to review randomisations and prioritise workload and progressively action outstanding issues.

### Plans for communicating important protocol amendments to relevant parties (e.g. trial participants, ethical committees) {25}

Protocol amendments will be agreed among the trial management group then categorised by sponsor before being reported for approval to the Research and Ethics Committee.

### Dissemination plans {31a}

The trial results will be communicated through presentation at conferences and publications, through professional, patient, and public outlets and via social media.

## Discussion

REBOA has the potential to improve outcomes in exsanguinating trauma patients. However, its role remains to be defined. This pragmatic, multi-centre, prospective, randomised clinical trial has been designed to establish the clinical and cost-effectiveness of the technique.

REBOA is a complex intervention, and its evaluation is therefore challenging. The UK-REBOA trial remains the only randomised study of the technique, worldwide. Aspects of the design which are likely to prove particularly problematic included the small number of eligible patients, the inclusion criteria, the implementation of the technique, enrollment and early data collection, and the ethical obligations associated with a research-without-prior-consent study.

The issue of a small eligible patient population was addressed with a Bayesian analytical framework. The output of a Bayesian analysis is the actual probability of a benefit, rather than a *p*-value [40, 41]. This helps to avoid the dichotomisation of trials into “significant” and “not significant,” which is widely regarded as unhelpful. A 75% probability of a mortality benefit, for example, might be sufficient to convince clinicians (and patients) that REBOA is a useful technique. Bayesian frameworks also offer more flexibility when it comes to formulating meaningful decision criteria, such as when to declare a trial successful, or when to stop a trial early. In conjunction with a range of prior probability distributions, these designs can also provide more nuanced interpretations. As a result, Bayesian trial designs are increasingly used, and the Food and Drug Administration has provided specific guidance regarding their role in medical device trials [42].

There are no agreed criteria (such as blood pressure, or transfusion threshold) to guide clinicians when REBOA should be considered. Outside the context of a trial, the decision to use the technique is based on a global “Gestalt” assessment, which includes multiple clinical parameters (blood pressure, response to transfusion, injury pattern, lactate, and base deficit), bedside investigations (Focused Assessment with Sonography for Trauma scanning, X-ray) and temporal factors such as time from injury to arrival in hospital/assessment. In line with the pragmatic design, the UK-REBOA trial aims to emulate this type of decision-making, which has been shown to be as accurate as more “objective” criteria [43]. The inclusion criteria were selected accordingly, to promote the recruitment of a range of participants, and to ensure the generalisability of the findings.

Sites participating in the trial, most of whom had not previously used the technique, are provided with extensive training both prior to starting enrolment, and subsequently. Our intention is to facilitate the development of a sustainable local “REBOA service”, which ensured that providers were adequately trained and credentialled.

Given that patients who require REBOA may present at all hours, and with very little notice, we devised an innovative, web-based mechanism for enrolling and randomising patients, and collecting essential data. The trial website ensured that patients could be enrolled without dedicated research staff, while also not interfering with the care of severely injured patients at immediate risk of dying. Furthermore, the majority of data points required for the trial are already routinely collected, minimising duplication, and easing the workload of research staff.

The pragmatic design does not, however, mandate specific actions, during the patient’s initial resuscitation, or subsequently. Given the heterogeneity of trauma patients and their injuries, this would be difficult to implement. In addition, the relatively small size of the trial, and the resulting credible intervals, may result in findings which are difficult to interpret. Furthermore, the choice of primary outcome (mortality at 90 days), which is intended to capture possible late detrimental effects of the technique, was made prior to research becoming available which suggests that haemorrhage control interventions should be evaluated with early mortality time points [34, 44, 45]. However, these have been included as secondary outcomes.

In conclusion, the UK-REBOA trial is a randomised, controlled, pragmatic trial that will provide the most definitive comparative data to date on the effectiveness of the procedure in reducing mortality in exsanguinating trauma patients.

## Trial status

Recruiting commenced on 1 January 2018. Enrollment is currently timetabled to end in June 2022. The current version of the protocol is Version 6 (25 November 2021).

## Supplementary information

**Additional file 1.** Consent forms.

## Abbreviations

ADE: Adverse device event
AE: Adverse event
CHaRT: Centre for Healthcare Randomised Trials
CTIMP: Clinical Trial of Investigational Medicinal Products
DMC: Data monitoring committee
eCRF: Electronic case report form
HES: Hospital Episode Statistics
HTA: Health Technology Assessment
NIHR: National Institute for Health Research
OR: Odds ratio
PI: Principal investigator
PROM: Patient-reported outcome measures
QALY: Quality-adjusted life years
ONS: Office of National Statistics
REBOA: Resuscitative endovascular balloon occlusion of the aorta
SADE: Serious adverse device event
SAE: Serious adverse event
SAP: Statistical analysis plan
TARN: Trauma Audit and Research Network
VOI: Value of information
