# Supplementary material for: The effectiveness and cost-effectiveness of resuscitative endovascular balloon occlusion of the aorta (REBOA) for trauma patients with uncontrolled torso haemorrhage: study protocol for a randomised clinical trial (the UK-REBOA trial)
Source: Trials. 2022 May 12;23:384. doi: 10.1186/s13063-022-06346-1 (PMC9097076; doi:10.1186/s13063-022-06346-1)
Supplement: Supplementary file 1 — Additional file 1. Consent forms. [file 13063_2022_6346_MOESM1_ESM.docx]

# Appendix: Consent forms
